# Oral Health Conditions and Domain-Specific Cognitive Decline in Older Adults: BLSA 2004-2024

**DOI:** 10.1093/geroni/igaf122.1224

**Published:** 2025-12-31

**Authors:** Xiang Qi, Huabin Luo, Bei Wu

**Affiliations:** New York University, New York, New York, United States; East Carolina University, Greenville, North Carolina, United States; NYU Shanghai, Shanghai, China (People’s Republic)

## Abstract

Associations between poor oral health and cognitive impairment have been documented, yet research examining associations between various oral health conditions and domain-specific cognitive decline remains limited. Using data from the Baltimore Longitudinal Study of Aging (2004-2024), we investigated the association between cognitivelymultiple oral health conditions and cognitive decline across domains in adults aged 50+ who were cognitive intact at baseline. Cognitive function was assessed across language, memory, attention, executive function, and visuospatial ability domains, with domain scores calculated as means of standardized cognitive test scores. Oral health was evaluated through objective assessment of tooth loss and dental plaque, alongside self-reported periodontal symptoms at baseline. The study included 980 participants (49.3% women, 66.5% White) with a mean age of 68.2 years (SD = 10.1), followed for about 7.8 years (SD = 4.9). Significant tooth loss showed associations with greater declines in global cognitive function (β=-0.0024; 95% CI: -0.0037, -0.0010), and across all cognitive domains, including language (β=-0.0035; 95% CI: -0.0049, -0.0020), memory (β=-0.0041; 95% CI: -0.0060, -0.0021), attention (β=-0.0028; 95% CI: -0.0045, -0.0011), executive function (β=-0.0018; 95% CI: -0.0032, -0.0003), and visuospatial ability (β=-0.0037; 95% CI: -0.0052, -0.0021). Moderate/significant dental plaque was associated with declining in global cognitive function (β=-0.0041; 95% CI: -0.0075, -0.0008) and memory (β=-0.0051; 95% CI: -0.0097, -0.0005), compared with no/hardly visible plaque, while moderate/severe periodontal symptoms solely predicted executive function decline (β=-0.0013; 95% CI: -0.002, -0.0003). Poor oral health significantly impacts memory and executive function, with tooth loss showing broader cognitive effects compared to other oral health conditions.

